# Leaffooted bugs enrich local soil with their horizontally acquired symbiont

**DOI:** 10.3389/fmicb.2026.1737071

**Published:** 2026-06-08

**Authors:** Bibek Singh Parajuli, John Teodosio, Alison Ravenscraft

**Affiliations:** Department of Biology, The University of Texas at Arlington, Arlington, TX, United States

**Keywords:** *Caballeronia*, horizontal transmission, host–microbe interaction, *Leptoglossus*, moisture, symbiont fitness, symbiosis

## Abstract

Associations between hosts and their microbial symbionts are considered mutualistic when both partners benefit. While the advantages received by eukaryotic hosts from association with bacterial symbionts are frequently examined, benefits to the bacteria are rarely experimentally tested. Here, we consider whether the bug-*Caballeronia* symbiosis is truly mutualistic by measuring the effect of a leaffooted bug (*Leptoglossus phyllopus*) on the abundance of its horizontally acquired symbiont, *Caballeronia grimmiae*. We predicted that the free-living *Caballeronia* population would increase over time in the presence of its insect partner. We quantified *Caballeronia* titer in soil microcosms (i) at different bug densities, and (ii) in the presence and absence of *L. phyllopus* over time. As bug density increased, the soil *Caballeronia* population also increased. Insect presence resulted in a marginally higher *Caballeronia* titer over time. Additionally, *Caballeronia* titer tended to be higher in wetter soil, though this correlation should be verified by follow-up work. These results suggests that the relationship between *Caballeronia* and *L. phyllopus* is likely mutualistic and add to a small but growing body of literature that has quantified the effects of eukaryotic hosts on their bacterial partners.

## Introduction

Most multicellular organisms associate with microbial symbionts (McFall-Ngai, 2014; Moran, 2007). These associations have played a crucial role in the evolution of the eukaryotic cell, evolutionary diversification, and the development of novel ecological innovations (Gerardo et al., 2020; López-García et al., 2017; McFall-Ngai, 2014; Moran, 2007; Sudakaran et al., 2017). Among the animals, insects are unique for their documented taxonomic and functional diversity of microbial symbionts (Engel and Moran, 2013; Moya et al., 2008). Benefits provided to insect hosts include nutrient synthesis, digestion of recalcitrant compounds, protection against pathogens and parasites, and increased tolerance to environmental stressors such as desiccation or extreme temperatures (Douglas, 1998, 2009; Engl et al., 2018; Flórez et al., 2015; Lemoine et al., 2020).

Insect-microbe symbioses are usually assumed to be beneficial for both partners, but most investigations focus on benefits to the host. Relatively few studies have measured *host* contributions to *symbiont* fitness (reviewed in: Garcia and Gerardo, 2014; Hoang et al., 2024; Mushegian and Ebert, 2016; Wooldridge, 2010). Many researchers fail to consider alternative non-mutualistic possibilities (Mushegian and Ebert, 2016). Labeling an interaction mutualistic before the net outcome for both partners has been quantified may lead to incorrect assumptions about the interaction’s ecological and evolutionary consequences (Bronstein, 2001; Mushegian and Ebert, 2016).

Many of the benefits a microbe might receive ultimately depend on how tightly it associates with the host. Some microbes are obligately associated with insects and cannot survive separately. They are reliably transmitted from parent to offspring (vertical transmission), or between host individuals (horizontal transmission) via processes including trophallaxis (e.g., oral-oral or oral-anal feeding in social insects; Bright and Bulgheresi, 2010; Salem et al., 2015). The advantages these obligately associated microbes receive are usually measured in terms of immediate physiological benefits, such as nutrients provided by the host (Bennett and Moran, 2015; Douglas, 1998; Fisher et al., 2017; Moran et al., 2008; Salem et al., 2015), or benefits to the host population itself, since the symbiont population is inseparable from the host population. Other microbes facultatively associate with insects and are capable of living independently (Salem et al., 2015; Wollenberg and Ruby, 2012). In addition to measuring the physiological benefits received within the host, investigators must also consider the net effect of host association on these microbes’ free-living population.

Within the host, a free-living symbiont can experience both beneficial and adverse physiological conditions. The potential benefits include a stable environment within the host, freedom from competition and predation, dispersal, and nutrient provisioning to the symbiont (Garcia and Gerardo, 2014). However, these benefits are not guaranteed (Garcia and Gerardo, 2014). Polyclonal symbiont populations are common in host-microbe associations, potentially leading to intense competition within the host (Baker and Romanski, 2007; Dubilier et al., 2008; Gage, 2002; Garcia et al., 2014; Martens et al., 2003). For example, in the *Wolbachia-* braconid wasp (*Asobara tabida*) symbiosis, one *Wolbachia* genotype showed decreased abundance in the presence of competing strains (Mouton et al., 2004). In *Riptortus pedestris*, the *α*-*Caballeronia* subclade outcompetes other *Caballeronia* subclades in the host insect’s midgut, despite equal ability to colonize the insect when infected in monoculture (Lextrait et al., 2025). Moreover, while symbionts may receive protection from predation within the host, they are also subjected to the immune system (Kim et al., 2013a; Login et al., 2011; Ratzka et al., 2013). Evidence suggests that in at least some symbioses, the host suppresses the symbiont population (Byeon et al., 2015; Kim et al., 2013a; Login et al., 2011; Park et al., 2018; Ratzka et al., 2013). Furthermore, although hosts can provide nutrients such as amino acids, sugars, and a carbon source to their symbiotic partner (Graf and Ruby, 1998; Ohbayashi et al., 2019; Zúñiga et al., 2018), studies showing that symbionts receiving host-derived nutrients perform better than their free-living counterparts are rare. Whether, and how often, host-provided benefits outweigh the physiological costs of host association and lead to net benefit to the free-living symbiont are open questions.

A few prior studies have measured how hosts affect the size of their symbiont’s free-living population. Garcia et al. (2019) tested how presence of a social amoeba, *Dictyostelium discoideum,* affected the population of two facultative symbionts, *Paraburkholderia agricolaris* and *P. hayleyella.* When the host was present, *P. agricolaris* growth was unchanged, but the abundance of *P. hayleyella* increased. In the squid-*Vibrio* symbiosis, *Vibrio* cells proliferate inside the host using host-derived nutrients and are subsequently expelled, augmenting the abundance of symbiotic *Vibrio* in the surrounding water (Graf and Ruby, 1998; Lee and Ruby, 1994; Wollenberg and Ruby, 2012). *Drosophila* fruit flies excrete N-acetyl-glucosamine and other nutrients that increase the abundance of their gut symbiont, *Lactobacillus plantarum*, in the surrounding medium (Storelli et al., 2018). Upon the death of hydrothermal vent tubeworms, their endosymbiont, *Candidatus* Endorifita, rapidly escapes the worm and reenters the environment (Klose et al., 2015). The effect of host association on the fitness of free-living symbiont populations has been studied in greater detail in the horizontally transmitted legume-rhizobia symbiosis (Burghardt, 2020; Denison and Kiers, 2011; Masson-Boivin and Sachs, 2018). Within legume nodules, rhizobia cells proliferate, aided by host-provided nutrients, and are eventually released from senescing nodules into the soil. An estimated 10^8^ descendant cells can be released into the soil from a single nodulating rhizobia cell (Denison and Kiers, 2011; Nuc and Olejnik, 2025; Udvardi and Poole, 2013). Indeed, several studies have shown an increased soil rhizobia population in the presence of a legume host (Brockwell et al., 1987; Denison and Kiers, 2011; Geremu et al., 2025; Kuykendall, 1989; Thies et al., 1995). This work indicates that there are at least three mechanisms by which a host can augment its symbiont’s free-living population: release of nutrients or direct release of live symbiont cells into the environment, either in feces or upon host death. In all cases, increased density of the host should result in elevated local density of the free-living symbiont.

The environmentally acquired bug-*Caballeronia* symbiosis offers an opportunity to measure the net benefit (or cost) of host association for a microbial partner. Thousands of species of true bugs in at least 7 taxonomic families acquire *Caballeronia* bacteria from the soil every generation as young nymphs during their second instar (Kikuchi et al., 2007, 2011). The symbiont colonizes a specialized section of the midgut, the M4 region, which is dedicated to housing the symbiont (Itoh et al., 2019; Kikuchi et al., 2005, 2011; Ohbayashi et al., 2015; Xu et al., 2016). *Caballeronia* recycles host nitrogenous waste to produce amino acids and vitamins which the insect obtains by digesting excess symbiont cells in the anterior midgut (Ohbayashi et al., 2019; Byeon et al., 2015; Futahashi et al., 2013). This promotes faster insect development, lower mortality, and larger body size (Acevedo et al., 2021; Garcia et al., 2014; Hunter et al., 2022; Kikuchi et al., 2007; Ravenscraft et al., 2020; Stoy et al., 2023). Transcriptomic data suggest that *Caballeronia* receives sugars (ribose, rhamnose), nitrogenous wastes (allantoin, urea), and sulfonates (taurine, alkanesulphonate) as nutrients from the insect (Ohbayashi et al., 2019), and the *in vivo* titer of *Caballeronia* increases as the insect ages (Kim et al., 2014; Stillson et al., 2025). However, evidence suggests that *Caballeronia* experiences nutrient limitation in the M4, as well as exposure to antimicrobial peptides which the host uses to control its population (Byeon et al., 2015; Itoh et al., 2019; Kim et al., 2013b; Lachat et al., 2024; Lee et al., 2023). Adults of some bug species can release live symbiont cells in their feces (Villa et al., 2023), but the effect on the free-living symbiont population is unknown.

We measured the effect of presence and density of the eastern leaffootted bug, *Leptoglossus phyllopus* (Coreidae), on *Caballeronia*. First, we quantified the abundance of *Caballeronia* in soil exposed to different host densities (0, 5, 15, and 30 bugs), predicting that increased host density would lead to higher *Caballeronia* soil titer. In a second experiment, we compared soil abundances of *Caballeronia* in the presence and absence of *L. phyllopus* over time, predicting that the insect would augment the free-living *Caballeronia* population such that the difference between the “insect present” and “insect absent” treatments would increase over time. This study furthers our understanding of the fitness outcomes of host–microbe interactions and, thus, the conditions necessary for the persistence of these interactions.

## Methods

### *Leptoglossus phyllopus* rearing

The eastern leaffooted bug, *Leptoglossus phyllopus,* ranges from the eastern and southern United States down into central Mexico. It feeds on a wide range of seeds and fruits and can be a pest on crops including tomatoes, peppers and oranges (Mitchell, 2006). We obtained insects from a laboratory colony at the University of Texas at Arlington. The colony was established from wild individuals collected in an urban lot in Arlington in 2019. The colony was maintained in mesh cages with bush bean plants (*Phaseolus vulgaris*) and fed raw Spanish peanuts. Cages were maintained in a VIVOSUN Grow Tent (Ontario, CA) set at ~28 °C with a 16:8 light:dark cycle. Males and females were paired to produce eggs, which were transferred to a screened plexiglass box to hatch.

### Experiment 1: soil *Caballeronia* abundance at different bug densities

**Figure 1:**
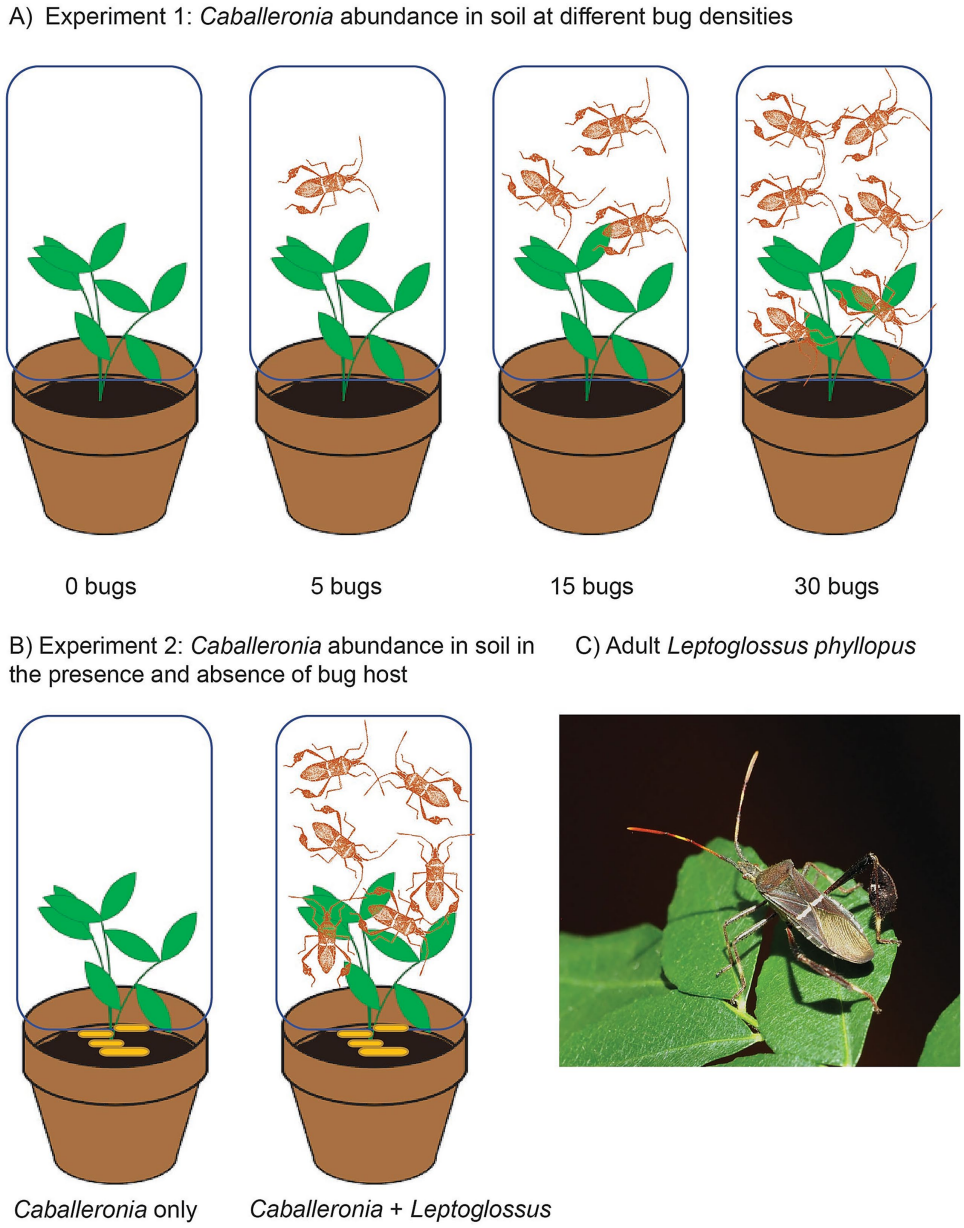
Outline of methodology. **(A)** In experiment 1, we calculated the change in *Caballeronia* abundance over 1 month in soils exposed to different bug densities (0, 5, 15, and 30 bugs/microcosm). **(B)** In experiment 2, we compared soil *Caballeronia* abundance in the presence and absence of bugs. An equal number of *Caballeronia* was added to both treatments at the beginning of the experiment. In the insects-present treatment, we added 10 nymphs to each enclosure each month. We collected soil samples for 5 months. **(C)** Adult *Leptoglossus phyllopus*.

We constructed soil microcosms using ~0.7 liters of Miracle-Gro moisture-control potting mix in 4 inch x 3–1/2 inch pots, topped with one-gallon plastic jars with a ventilated side (Figure 1A). Peanuts attached to the side of the enclosures were provided as food. Cowpea cuttings (*Vigna unguiculata*) in floral vials were placed inside the enclosures as a source of water and shelter. The enclosures were maintained under the same conditions as the colony cages mentioned above.

To determine whether the free-living *Caballeronia* population increases with insect density, we measured symbiont abundance in microcosms exposed to different insect densities. Soil in this experiment was sterilized via autoclaving at 121 °C for 30 min and no symbiont cells were added; insects were therefore the only source of *Caballeronia*. Experiment 1 was designed to focus on the contribution of symbiont-colonized insects to soil titer (whereas in Experiment 2, we allowed aposymbiotic nymphs to acquire *Caballeronia* from soil as they would naturally).

*L. phyllopus* nymphs were colonized with *Caballeronia grimmiae*, Lep1A1 cells cultured at 28 °C in YG broth overnight, shaking at 270 rpm (Hunter et al., 2022; Stillson et al., 2022). This strain was isolated from a laboratory colony of *Leptoglossus zonatus* at the University of Arizona, derived from insects originally collected in California, and is common in wild bugs (Ravenscraft et al., 2024). To obtain log-phase cells, 500 μL of the overnight culture was transferred to 2.5 mL of fresh broth and cultured for several hours. Log phase culture was diluted to an OD of 0.1 and fed to freshly molted second instar nymphs for 3 days. Nymphs were then introduced into enclosures at different densities (0, 5 bugs, 15 bugs, and 30 bugs; Figure 1A). Enclosures were maintained in the VIVOSUN tent for 33 days. Soil moisture and bug density were monitored weekly. All nymphs had either developed into adults or died by the end of the experiment. The dead nymphs were left in the microcosms to decompose. Soil samples (~100 mg) were collected from each enclosure at the beginning and end to quantify *Caballeronia.*

Additionally, at the end of the experiment, one soil sample per enclosure was treated with PMAxx™ (Biotium, California, US) to determine whether the *Caballeronia* cells released by the bugs were viable. Detailed descriptions of the PMA-qPCR technique, along with the optimization, protocol parameters, and statistical analysis, are presented in Supplementary Appendix A.

### Experiment 2: soil *Caballeronia* abundance in presence and absence of host

We constructed enclosures as described in Experiment 1. The soil in this experiment was not autoclaved (unlike Experiment 1). Soil was inoculated with *C. grimmiae* LepA1. A single colony was used to inoculate 70 mL of yeast-glucose (YG) broth, which was incubated overnight at 28 °C with shaking at 270 rpm. The overnight culture was diluted to an optical density (OD) of 0.8 using sterile water.

The “host” present versus “host” absent design was informed by prior studies that have quantified the effect of host on free- living symbiont population (Garcia and Gerardo, 2014; Garcia et al., 2019; Lee and Ruby, 1994) (Figure 1B). For both treatments (“insect absent” and “insect present”), the soil in every enclosure was initially sprayed evenly with 2.2 mL of this cell suspension, which covered a surface area of 12.6 inches^2^. Each treatment had 15 replicate enclosures. In nature, aposymbiotic nymphs acquire their *Caballeronia* by ingesting it from soil (Kikuchi et al., 2007). Experiment 2 was intended to mimic a scenario in which aposymbiotic nymphs arrive in an environment where *Caballeronia* is readily available (unlike in Experiment 1, where the nymphs were preinfected with *Caballeronia* before they were introduced into the enclosures).

All enclosures were maintained in the VIVOSUN grow tent for 7 months. In the *“*insect present” treatment, 10 first or second-instar nymphs were placed inside each enclosure once per month. Because we observed high mortality in the second batch of added nymphs, we re-inoculated all microcosms with *Caballeronia* on day 76, which reset the *Caballeronia* titer in soil to Day 2 levels (Supplementary Figure S2). Some, but not all, nymphs we added to the experiment after the reinoculation reached the adult stage. To reduce crowding, we removed eggs laid by the bugs; however, some eggs escaped our detection, resulting in accumulation of additional insects within the “insect present” enclosures. The total number of bugs in each enclosure fluctuated throughout the experiment; most of the time, there were between 4 and 17 insects per enclosure. Dead nymphs and adults were left in the enclosures to decompose.

Soil samples (~200 mg) were collected in 1.7 mL microcentrifuge tubes at 2, 63, 78, 90, 108, 138, 200, and 215 days post-inoculation and held at −80 °C. A spatula was used to mix the topsoil prior to sampling in an attempt to distribute the *Caballeronia* more evenly. Since the soil was reinoculated on day 76, the soil collected beyond this timepoint was relabeled as 2, 14, 32, 62, 124, and 139 days post-reinoculation (dpr). Unless otherwise stated, all the timepoints mentioned hereafter are days post-reinoculation.

Prior to sample collection, we measured soil moisture using a Dr. Meter soil moisture meter© (Hong Kong), which ranges from 1 (driest) to 10 (wettest). While we attempted to maintain moderate and equal soil moisture throughout the experiment, soil moisture did vary over time, with soil drying somewhat between waterings. The soil in the microcosms was watered twice a week until the soil moisture was within the 4–6 range, as measured with the moisture meter. We used this variation to test the correlation between soil moisture and *Caballeronia* abundance. The soil moisture levels in the enclosures were measured over time, but were not experimentally manipulated. Over 85% of the moisture measurements were within the “moist” 4.0–6.5 range at the time of soil sampling. The occasional extreme measure was no lower than 3.5 (still damp), and the occasional high was never above 8 (wet but not saturated). The soil moisture meter operates on the principle of galvanic cell-based sensor and measures relative wetness based on electrical conductivity rather than the actual volumetric water content in the soil. The relation between electrical conductance and volumetric water content is approximately linear at lower moisture levels and nonlinear at higher moisture levels (Kivimeister et al., 2026).

### DNA extraction and qPCR

DNA was extracted from the soil samples using Quick-DNA™ Fecal/Soil Microbe Miniprep kit (Zymo Research) following the manufacturer’s protocol for soil, except that we used a TissueLyser II at 25.0 Hz for 7 min to homogenize the samples instead of the proprietary bead beater. Additionally, to improve DNA yield, we added 800 μL of Genome lysis buffer and 400 μL of 100% ethanol to the filtrate from the Zymo-Spin III-F filter.

To quantify symbiont titer, we designed species-specific primers using the genome of *C. grimmiae* Lep1A1. We identified a single copy gene unique to this species (Stillson et al., 2022) and used PrimerQuest™ Tool (IDT) to design primers qdhm-F (5’-TTGCGA CCTGTTCCTTTCA-3′) and qdhm-R (5’-CGTGTGATAGTCG CCGTTAT-3′), which target a 151-bp region of the dihydromethanopterin reductase (DHMR) gene. NCBI BLAST predicted *C. grimmiae* Lep1A1 to be the sole organism amplified by these primers, and we experimentally verified primer specificity by running diagnostic PCR with several phylogenetically distinct strains of *Caballeronia* (LP003, LZ003, LZ029) and a closely related *Cupriavidus* strain (LZ004). We tested a gradient of annealing temperatures from 60 to 68 °C and the temperature that produced the brightest band (60 °C) was used.

To create standards for absolute quantification of DHMR copy number, we amplified Lep1A1 genomic DNA with the qdhmR/F primers. The concentration of the amplified PCR product was determined using a Qubit dsDNA High-Sensitivity Assay (ThermoFisher Scientific, Waltham, MA, United States). We calculated the number of DHMR copies using the DNA Copy Number and Dilution Calculator (ThermoFisher Scientific). We made 10-fold dilutions ranging from 10 copies per μL to 10^7^ copies per μL.

The qPCR assays were run on an Applied Biosystems 7,300 Real-Time PCR System (ThermoFisher Scientific). Each reaction contained 10 μL PowerUp SYBR Green master mix (Applied Biosystems, Waltham, Massachusetts), 4 μL of molecular-grade water, 2 μL each of forward (500 nM) and reverse primers (500 nM), and 2 μL of template DNA. The thermocycling program was 50 °C for 2 min to activate uracil-DNA glycosylase (which prevents reamplification of carryover PCR products), 95 °C for 2 min, followed by 40 cycles of 95 °C for 15 s and 60 °C for 1 min. To determine if non-specific products were amplified, we finished with a melt curve ramping from 60 °C to 95 °C. qPCR replicates that produced non-specific amplification, as indicated by the melting temperature, were removed from downstream analysis. Each soil sample was run in triplicate and replicates were averaged. No template controls were included on every run. Amplification efficiency ranged from 85 to 114% across all runs.

### Analysis

In our first experiment, we used a linear regression model (lm command in R) to determine the effect of bug density on the *Caballeronia* population after a one-month exposure to insects. Independent variables were bug density and bug density squared (to allow for a non-linear relationship). The dependent variable was the change in *Caballeronia* DHMR copy number from the initial time point (0 days) to the final time point (33 days). Prior to calculating the difference, we applied a ln(mean + 1) transformation to DHMR copy number to meet the normality assumption of linear mixed-effect models and deal with zero values.

For the second experiment, we analyzed the effect of *L. phyllopus* presence on the abundance of *Caballeronia* in soil microcosms over time with linear mixed effect models (LMM) using the lmer command in R. A full model was run with fixed terms for treatment (insects present or insects absent), number of days post soil inoculation (time), scaled squared days post inoculation (time^2^) to account for a nonlinear relationship, and soil moisture, as well as all pairwise interactions between these terms except for the time x time^2^ interaction. Microcosm was accounted for as a random effect. We performed backward model selection with likelihood ratio tests to determine the final model. Model residuals were tested for normality and homoscedasticity using the Shapiro–Wilk test and by plotting the residuals against the predicted values, respectively. *Caballeronia* abundance was log-transformed to meet the normality assumption of linear mixed-effect models.

## Results

### Experiment 1: *Caballeronia* abundance in soil increases with bug density

**Figure 2:**
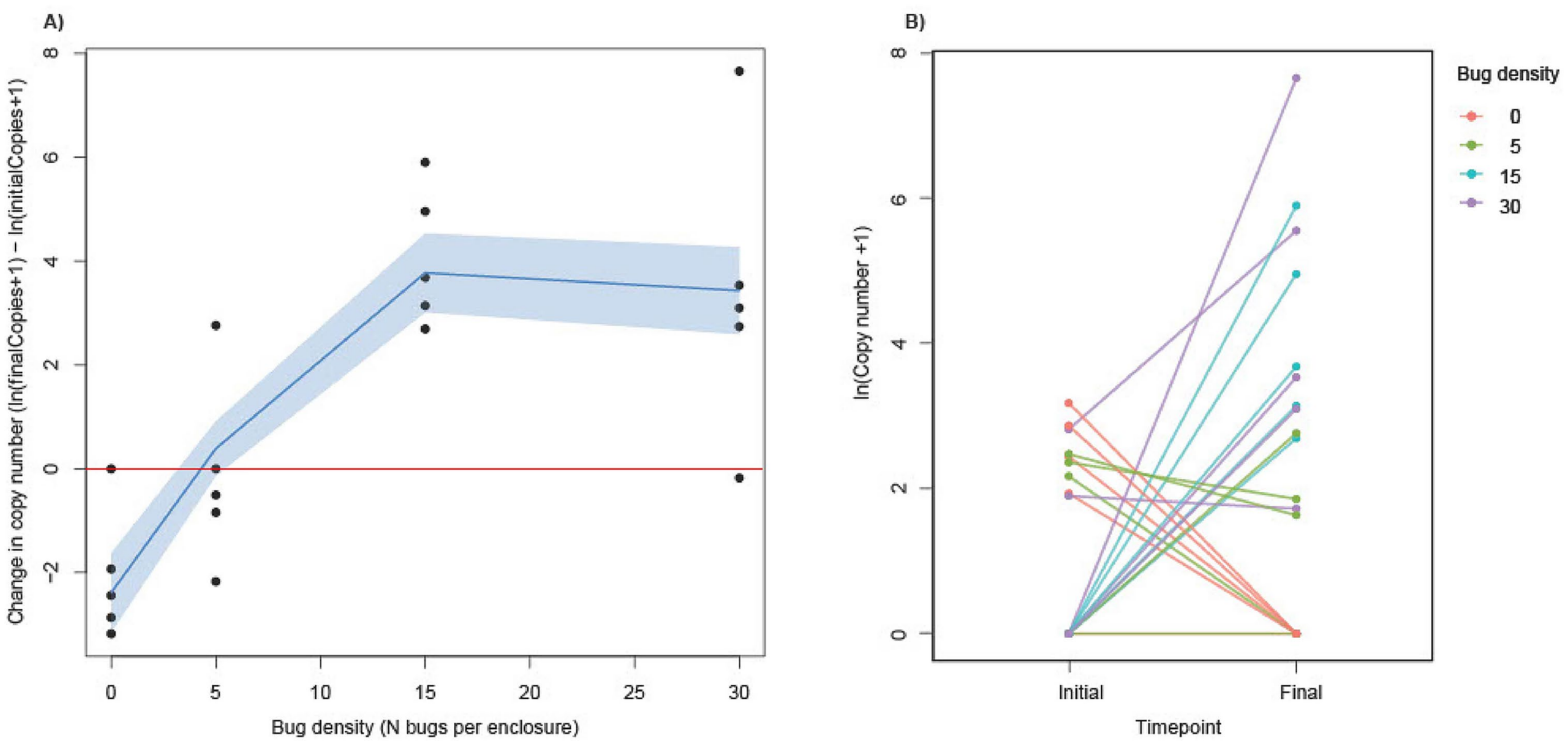
**(A)** Effect of host density on change in *Caballeronia grimmiae* abundance in soil over 1 month. The blue line shows the model estimated mean difference and blue shading depicts the estimated standard error. Each data point is an observed value (difference between the means of triplicate titer measurements). **(B)** *Caballeronia* abundance (log-transformed mean copy number +1) in soil at the beginning and at the end of the experiment. Each data point is an observed value for an individual replicate enclosure, with the lines showing the trajectory of the *Caballeronia* over 1 month. Color indicates the bug densities.

First, we tested whether higher insect density promotes higher *Caballeronia* abundance in soil. For this experiment, we used autoclaved soil and only the insects (not the soil) were inoculated with *Caballeronia*. Over the course of 1 month, *Caballeronia* titer remained essentially unchanged in insect-free microcosms, but increased with rising insect density (Figure 2A; bug density: df = 1, χ^2^ = 68.874, *p* < 0.001; bug density^2^: df = 1, χ^2^ = 36.928, *p* = 0.002). The benefit to *Caballeronia* showed diminishing marginal returns, reaching a plataeu by 15 bugs per microcosm. At 15 bugs per microcosm, the average one-month increase in *Caballeronia* abundance was 1,160 additional copies per gram of soil (Figures 2A,B). To ascertain whether *Caballeronia* were viable, we collected duplicate soil samples from each microcosm at the end of the experiment and treated these with PMAxx, which prevents amplification of DNA from dead cells. Estimates of live *Caballeronia* density (PMAxx-treated soils) were higher than estimates of total *Caballeronia* density in the same soils (without PMAxx treatment) in the 0 bug and 5 bug enclosures (see Supplementary Appendix A; Supplementary Figure S1; Treatment X Bug density interaction: *p* = 0.018; post-hoc pairwise comparisons: *p* < 0.01), but did not vary at 15-bug or 30-bug enclosures (Supplementary Figure S1, *p* > 0.05).

After 1 month, we detected zero *Caballeronia* in the absence of bugs (Figure 2B), resulting in a slight decrease from the average starting titer. We autoclaved the soil prior to the start of the experiment to kill live cells, but DNA from these killed cells could still have been present. By the end of the experiment, this dead DNA appears to have degraded, which may explain the small observed decrease in *Caballeronia* in the 0-bug treatment.

### Experiment 2: *Caballeronia Grimmae* abundance in soil over time trends higher in the presence of *Leptoglossus phyllopus*

We inoculated soil microcosms with cultured *Caballeronia* cells and compared the soil *Caballeronia* population with or without *L. phyllopus*. We predicted that insects would supplement the free-living symbiont population such that, over time, soil exposed to insects would contain more and more *Caballeronia* compared to unexposed soil. Initially, the *Caballeronia* population plummeted (Supplementary Figure S2), and the second batch of nymphs all died. Therefore, the soil was reinoculated with *Caballeronia* 76 days after the first inoculation. We assumed the high mortality of the second batch of nymphs was due to the nymphs’ failure to acquire the symbiont, resulting from a decline in the *Caballeronia* population. However, when we processed the samples and analyzed the data, we found that the drop in symbiont titer was similar at 63 days post-inoculation and 138 days post-inoculation (62 days post-reinoculation; Supplementary Figure S2). It is possible that the second batch of nymphs were infected with a pathogen or hatched from eggs laid by older females, as egg mortality has been shown to increase with the age of the female (Umanzor et al., 2025). All analyses were performed on data collected after the reinoculation.

**Figure 3:**
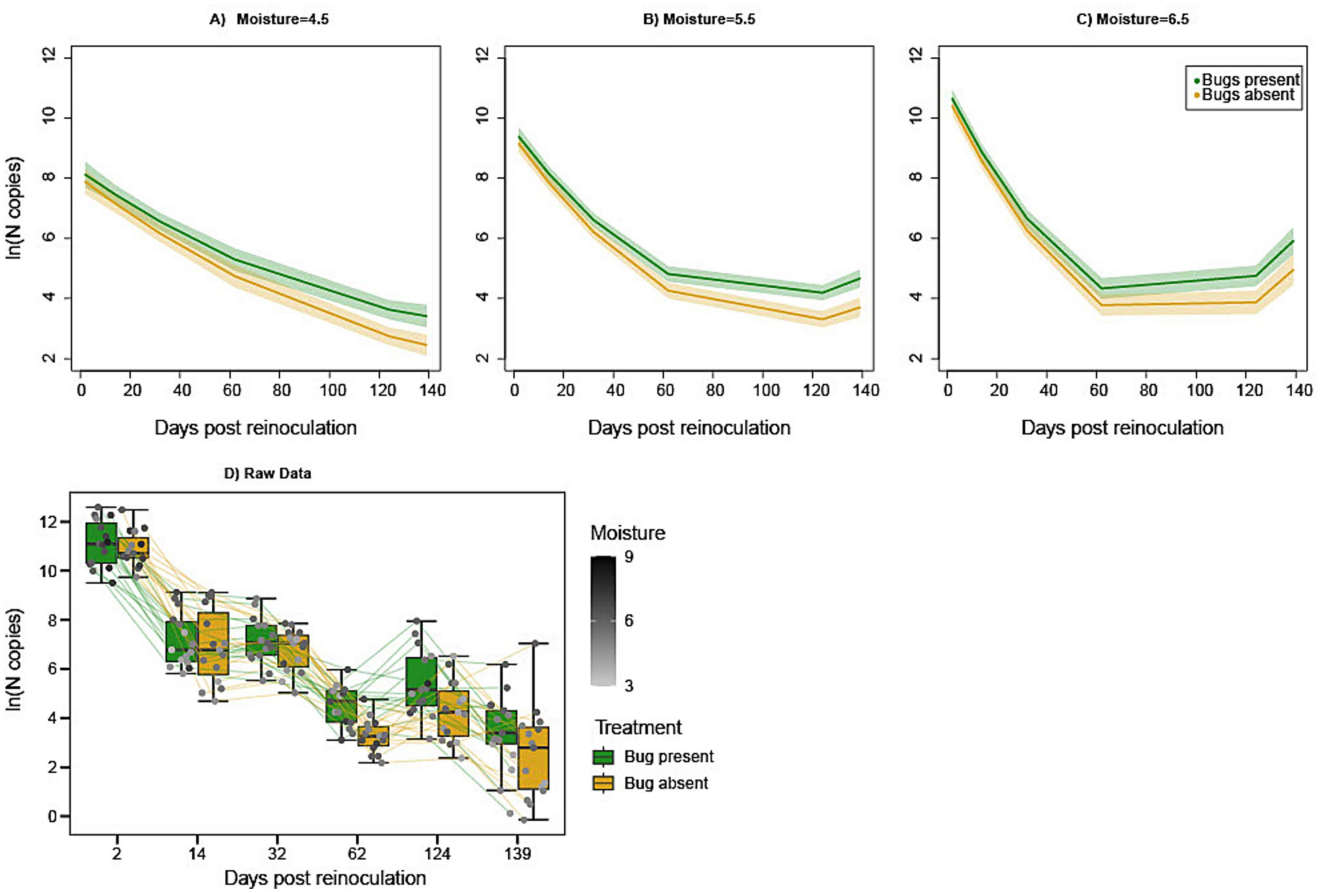
**(A–C)** Model-predicted effect of *Leptoglossus phyllopus* on *Caballeronia grimmiae* abundance in soil over time at different moisture levels. Moisture predictions are based on observed correlation between soil moisture and *Caballeronia* abundance, not experimental manipulation of soil moisture. Colored lines show model estimated means, and shading depicts estimated standard errors. **(A)** Moisture level = 4.5. **(B)** Moisture level = 5.5. **(C)** Moisture level = 6.5. **(D)** *Caballeronia* abundance in soil over time. Each point reports the mean of triplicate titer measurements, with lines tracing the trajectory of *Caballeronia* abundance in each microcosm over time. Y-axis reports the titer of *C. grimmiae* in 200 mg of soil. X-axis reports the days post reinoculation (dpr). Instances where soils were collected over 2–3 days have been set to a single day. The greyscale gradient on the individual data points represents the moisture level of the enclosure at the time of sampling.

Symbiont populations decreased in both treatments over the course of the 139-days post reinoculation, likely because our inoculations overshot the soil’s carrying capacity for *Caballeronia* (Figures 3A–C). Although the interaction between insect presence and time was not significant (treatment*time interaction term: df = 1, χ^2^ = 1.94, *p*-value = 0.16), we retained the interaction in the final model because omitting it resulted in flawed predictions. Specifically, the model with only main effects of treatment and time predicted that *Caballeronia* population would always be higher in soils exposed to insects, even at the beginning of the experiment, prior to insect introduction, and immediately after, when the soil was reinoculated. However, all soils were reinoculated identically, and we confirmed that *Caballeronia* populations were equal between microcosms with and without bugs before the reinoculation on day 63 (t-test, t (28) = −1.084, *p* = 0.2875), and also 2 days after we re-inoculated (t-test, t(28) = 1.037, *p* = 0.3086). Therefore, the increased abundance of *Caballeronia* in the presence of insects appears to have been driven by later time points. We think that the lack of significance of the interaction term was likely due to comparatively small sample size (15 microcosms per treatment) and high variance in *Caballeronia* abundance. We could not track where insects defecated or died, but these factors likely caused fine-scale but high-magnitude variation in *Caballeronia* density in the soil (to reduce this variation, the topsoil was mixed using a spatula prior to sampling.) We ran a power analysis using the powerSim function from the “simr” package, and it found we had only 30% power to detect an interaction effect of the observed magnitude.

We also found that higher soil moisture was correlated with larger *Caballeronia* populations. This relationship was nonlinear and depended on time, with *Caballeronia* population predicted to decrease to extinction in drier soil, but level off in wetter soil (Figures 3A–C; moisture*time interaction: df = 1, χ^2^ = 23.51, *p* < 0.001; moisture*time^2^ interaction: df = 1, χ^2^ = 24.13, *p* < 0.001). Interestingly, our model predicted that the *Caballeronia* population increases after day 124 in wetter soils. However, this predicted increase is questionable, as there was only one additional sampling point after this time. Soil moisture did not systematically differ between microcosms with and without bugs (model predicting moisture as a function of time and treatment), with random effect for microcosm: treatment term (df = 1, χ^2^ = 1.0233, *p*-value = 0.312). It should be noted that the soil moisture was not experimentally manipulated; rather, the correlation between soil moisture and *Caballeronia* titer was extracted from model predictions. We ran a single model on all observed data and plotted its predictions across a range of moisture levels to show the expected responses under different moisture conditions (Figures 3A–C).

At 62 days post-reinoculation and with an average soil moisture of 5.5, the model estimated there were about 382 more *Caballeronia* copies per gram of soil in microcosms with bugs than without bugs.

## Discussion

We found that both the presence of hosts over time and higher population density of leaffooted bugs were correlated with increased abundance of their bacterial symbiont in the environment. In the first experiment using uninoculated soil, *Caballeronia* abundance increased with insect density; the magnitude of increase started to plateau around 15 bugs and *Caballeronia* received diminishing marginal returns from additional insects. While we do not know whether the *Caballeronia* copies we detected in the soil were viable, we believe some of them were live cells. We used PMAxx™ (Biotium, California, US) to quantify live *Caballeronia* cells in Experiment 1. Although the method was imperfect, the results suggested that a meaningful number of the *Caballeronia* copies we detected may have derived from live cells (see Supplementary Appendix A; Supplementary Figure S1). In our second experiment, we lacked statistical power to detect a significant trend over time (treatment x time interaction, *p* = 0.16), but the direction of our results was consistent with our prediction.

Our findings suggest that the free-living *Caballeronia* population does benefit from association with *Leptoglossus*. The benefit to the *Caballeronia* could result from three mechanisms. First, bugs may excrete live cells in frass. Squash bugs (*Anasa tristis* and *Anasa andresii*), another leaffooted bug (Coreidae), release live *Caballeronia* in their feces (Villa et al., 2023). While we have not quantified the release of *Caballeronia* from *L. phyllopus*, we have previously isolated live symbiont from the insects’ feces, and live cells have been detected in the feces of the congener, *L. zonatus* (L. Sullivan, personal communication, February 2024). How long live cells excreted by these insects persist in the soil, and whether they establish a resident population, are relevant questions for future research.

Second, cells may escape from dead insect carcasses. In dead mammals, collapse of the immune system leads to rapid proliferation of the gut microbial population (Heimesaat et al., 2012), some of which may be released into the environment. Similar proliferation might occur in dead bugs, since production of the antimicrobial peptides used to control *Caballeronia* in the M4 (Byeon et al., 2015; Futahashi et al., 2013) should cease. (However, *Caballeronia* are aerobic and oxygen could rapidly deplete in a decomposing insect.) In both of our experiments, we left dead insects in the microcosm to decompose. Enrichment of the symbiont population via escape from dead hosts has been observed in tube worms: A medium-sized worm releases up to 7 × 10^5^ *Candidatus* Endoriftia symbionts into the environment upon its death (Klose et al., 2015).

Third, leaffooted bugs might release nutrients into the soil from frass or dead insect bodies, which can increase soil microbial biomass (Yang and Gratton, 2014). The deposition of dead cicada carcasses in forest plots increases soil nitrate and ammonium availability, leading to a higher relative abundance of soil bacteria (Yang, 2004). *Drosophila* larvae promote persistence of their facultative symbiont *Lactobacillus plantarum* in their shared habitat through intestinal secretions of the nutrient N-acetyl-glucosamine (NAG; Storelli et al., 2018). It is also possible that nutrients added to the soil from any insect (regardless of whether it hosts *Caballeronia*) could increase the abundance of *Caballeronia* in the soil. Whether cells or nutrients released from true bugs can similarly affect the population of free-living *Caballeronia* should be further investigated.

In addition to insect presence and density, soil physical and chemical properties can impact environmental bacterial abundances. Various studies have demonstrated that wetter soil conditions are correlated with an increased abundance and activity of soil microbial communities (Colombo et al., 2016; Manzoni et al., 2012; Morugán-Coronado et al., 2019; Zhang et al., 2013). Similarly, we found that moister soil was associated with higher *Caballeronia* abundance. Furthermore, the observed decline in symbiont population in dry soil suggests that insects may act as a refuge for *Caballeronia* in drier conditions. While the observed correlation suggests that soil moisture could be important for *Caballeronia*, our experiment was not designed to test for a causal relationship between soil moisture and symbiont titer. Further investigation on how soil moisture and chemistry (e.g., pH) affect host acquisition of *Caballeronia* will prove valuable in understanding how environmental conditions affect insect-microbe interactions.

Interestingly, in Experiment 2 *Caballeronia* abundance declined over time in both insect-present and insect-absent treatments (Figure 3), most likely due to our initial inoculation exceeding the soil’s carrying capacity. In dry soils, *Caballeronia* population trended toward extinction, but in wetter soils, the population appeared to reach their lowest point at approximately 193 cells per gram without insects and 483 cells per gram with insects on day 120 (Figure 3C). Studies evaluating the abundance of *Burkholderia* spp. in soil (many studies include *Caballeronia* as a *Burkholderia* spp.) have found a wide range, from 1 × 10^3^ colony forming units (CFU) per gram of dry soil to 3.18 × 10^7^ CFU/g dry soil (Draghi et al., 2018; Pallud et al., 2001; Salles et al., 2006). These studies used culture-based methods, which fail to detect certain species and likely underquantify *Burkholderia* abundance (Miller et al., 2002; Pirone et al., 2005). Using qPCR and MiSeq, one study directly quantified the natural abundance of *Caballeronia* in agricultural fields at 1.1 x $10^{7}$ copies per gram of soil (Tago et al., 2014). In summary, the variability in *Burkholderia* abundance across these studies is likely shaped by geographic variation, agricultural practices, and differences in soil physical and chemical conditions. Notably, we used commercial soilless potting mix while Tago and colleagues quantified *Caballeronia* in agricultural soil. Regardless, we still observed a marginal trend suggesting that insect presence augmented the titer of free-living *Caballeronia*, with the population exposed to bugs tending to decrease more slowly over time.

Only a handful of studies have measured how hosts affect the size of their symbionts’ free-living population. In the legume-rhizobium symbiosis, nitrogen-fixing bacteria are acquired from the soil and reproduce within root nodules, eventually escaping, repopulating the soil, and infecting future generations (Denison and Kiers, 2011). Indeed, one study found the soil rhizobial population to be five times larger in plots where leguminous plants were present (Kuykendall, 1989). In ocean environments, association with bobtail squid allows the bioluminescent bacterium *Vibrio fisheri* to grow to higher population sizes due to competition-free growth within the host’s symbiotic organ, followed by daily release of bacteria into the environment (Lee and Ruby, 1994; Wollenberg and Ruby, 2012). Enrichment of the local environment with symbionts should increase the likelihood that offspring find a symbiotic partner, while leaving ample opportunity for offspring to acquire other symbiont strains. This allows for the possibility that offspring acquire locally adapted strains that confer enhanced fitness under local conditions, while still bet-hedging against the risk that offspring acquire an inferior symbiont, or fail to acquire any symbiont (Bruijning et al., 2021; Lange et al., 2023; Stillson et al., 2025).

While we were primarily interested in the effect of the host on symbiont fitness, supplementing the free-living symbiont is also likely to benefit the host. In the first experiment, *Caballeronia* increased by an average of about 1,160 cells per gram in the presence of at least 15 bugs (Figure 2). In the second experiment, the final difference of ~1,100 cells per gram in the wettest soil represented a ~ 55% increase compared to soils unexposed to insects. These increases are likely meaningful for insect fitness, since in another *Caballeronia*-hosting bug, *Riptorus pedestris*, it took just 80 *Caballeronia* cells to infect an average of 50% of nymphs and 3,500 cells to infect 100% of nymphs (Kikuchi and Yumoto, 2013).

We studied the effect of the host on one symbiont strain, but host effects might vary across different host-symbiont pairings. For example, the advantage conferred by host association to microbial symbionts varies among symbiont species in the social amoeba-*Paraburkholderia* symbiosis*. Parabukholderia hayleyella* increased in population when associated with *Dictyostelium,* whereas *P. agricolaris* decreased when the host was present (Garcia et al., 2019). Different insect species might also have different effects on environmental symbiont abundance: While it seems likely that many coreid insects augment local populations of *Caballeronia* via release of cells in frass, the alydid bean bug (*Riptortus pedestris*) never sheds live *Caballeronia* in its frass (Kikuchi et al., 2007). These observations suggest that outcomes of the relationship between true bugs and the *Caballeronia* can vary with different combinations of host and symbiont strains. Additionally, the magnitude and direction (e.g., beneficial or detrimental) of these outcomes for one or both partners can shift based on biotic, abiotic, and stochastic contexts (e.g., priority effects; Chen et al., 2024a; Chen et al., 2024b; Leung and Poulin, 2008; Moxon and Kussell, 2017; Stillson et al., 2025).

## Conclusion

While the impact of microbial symbionts on host fitness has been the focus of most symbiosis research (Cani, 2018; Douglas, 1998; Kaltenpoth & Flórez, 2020; McFall-Ngai, 2002), benefits conferred to the symbiont are rarely quantified (Garcia and Gerardo, 2014; Hoang et al., 2024). Our data suggest that the population of free-living *Caballeronia* increases in the presence of its insect host, *Leptoglossus phyllopus*. Thousands of true bug species associate with *Caballeronia*; our findings suggest that these relationships can benefit the bacteria as well as the insects. Our results are consistent with similar studies of other environmentally acquired symbioses, including the squid-*Vibrio* luminescent symbiosis and the social amoeba-*Paraburkholderia* nutritional symbiosis (Garcia et al., 2019; Lee and Ruby, 1994). It is important to understand the effect of the multicellular hosts on their microbial partners to better understand the evolutionary and ecological dynamics underlying host-symbiont relationships.

## Data Availability

The datasets presented in this study can be found in online repositories. The names of the repository/repositories and accession number(s) can be found at: https://figshare.com/articles/dataset/Effect_of_Leptoglossus_phyllopus_on_Caballeronia_abundance/30034696.
