# Supplementary material for: Leaffooted bugs enrich local soil with their horizontally acquired symbiont
Source: Front Microbiol. 2026 Jun 8;17:1737071. doi: 10.3389/fmicb.2026.1737071 (PMC13284837; doi:10.3389/fmicb.2026.1737071)
Supplement: Supplementary file 1 [file Supplementary_file_1.docx]

Supplementary Material

**Supplementary Appendix A:** Use of PMAxx to quantify live *Caballeronia* cells in the soil at the end of Experiment 1.

An increasing number of studies have highlighted the importance of assessing bacterial cell viability to properly characterize and quantify the abundance of environmental microbial communities (Carini et al., 2016; Carini et al., 2020; Guo and Zhang, 2014; Nocker et al., 2025; Tekgul and Adiguzel, 2025; Thomas et al., 2025). Therefore, in an attempt to quantify only live, viable bacterial cells, we treated a set of soil samples from Experiment 1 with propidium monoazide (PMAxx™; Biotium, California, US), a high-affinity photoreactive DNA-binding dye that prevents amplification of DNA from membrane-compromised bacterial cells (Nocker et al., 2006).

PMAXX was designed for use with clear samples, but the manufacturer has also published recommendations for opaque samples. We followed these recommendations with slight modifications informed by previous studies (Fongaro et al., 2016; Guo & Zhang, 2014). Specifically, we treated each sample with 100 μM PMAxx (instead of the 25 μM recommended for clear samples), followed by incubation in the dark for 15 minutes (instead of the 10 minutes recommended for clear samples). Soil suspensions were then exposed to blue light for 30 minutes (instead of 15 minutes) with intermittent shaking to allow light to penetrate the samples and cross-link the PMAxx to dead DNA.

We first tested this modified protocol on soil spiked with a heat-killed overnight culture of *Caballeronia* Lep1A1. Half of this soil underwent a standard DNA extraction, while the other half was treated with PMAxx prior to standard DNA extraction. We then performed qPCR on the two extractions. The difference in threshold cycle (dCt) between the PMAxx-qPCR and standard qPCR for soil spiked with dead cells was 4.93, indicating that nearly 97% of the dead cells’ DNA was removed. The manufacturer recommends that the result for this dead-cell control is a dCT greater than 4. Our test therefore indicated that the PMAxx treatment was successful in inhibiting amplification of DNA from dead cells. Furthermore, the dCt between PMAxx-qPCR and standard qPCR for soil spiked with live cell culture was close to 0, indicating that the PMAxx did not suppress the amplification of viable cell DNA. We therefore applied this protocol to a set of our experimental samples. However, controls treated simultaneously with the experimental samples indicated that PMAxx treatment was not as effective as our initial testing (described below).

At the end of Experiment 1, one soil sample per enclosure was treated with PMAxx in an attempt to remove dead DNA and quantify only viable *Caballeronia*. DNA extraction and qPCR were then performed on these PMAxx-treated samples using the same protocol and parameters as described in the methods. Results are compared against the equivalent non-PMAxx treated samples in Supplementary Figure 1.


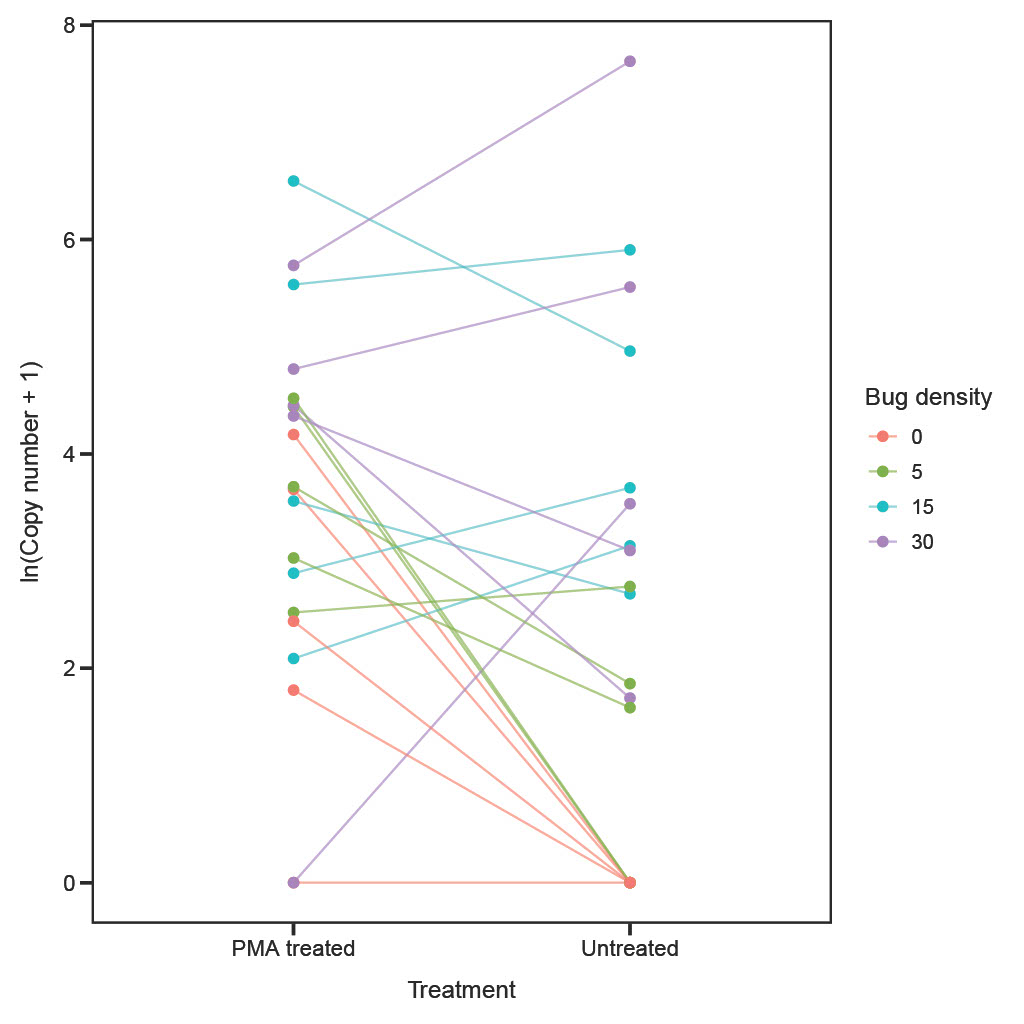


Supplemental Figure 1: Comparison between live and total *Caballeronia* titers in soil across different bug densities at the end of Experiment 1. Two soil samples were taken per microcosm and one from each set was treated with PMAxx to prevent quantification of DNA from dead *Caballeronia* cells. X-axis reports whether the soil at the end of the experiment was treated with PMAxx (to quantify live titers) or untreated (total live and dead) titer. Y-axis reports the *Caballeronia* titers (natural log of gene copy) per 100 mg of soil. Each data point represents an individual replicate enclosure, with lines connecting paired samples from each enclosure. Color coding indicates bug density (0,5,15, or 30)

We ran a linear regression to determine whether *Caballeronia* abundance differed between PMA-treated and untreated soil at the final time point across different bug densities. The dependent variable was the natural-log transformed mean DHMR copy number with a pseudo-count of 1 (ln (mean + 1)), and the independent variables were the treatment, bug density, and their interaction term. Following a significant interaction, we performed a post hoc pairwise comparison test using estimated marginal means.

Our estimates of live *Caballeronia* density (PMAxx-treated soils) were higher than total *Caballeronia* density in the same soils (without PMAxx treatment) (Fig. S1; Treatment X Bug density interaction: p = 0.018; post-hoc pairwise comparisons: p < 0.01) in the 0 bug and 5 bug enclosures, but did not significantly differ in 15-bug or 30-bug enclosures (p >0.05). While these results suggest that most of the *Caballeronia* we detected was likely viable, the higher abundance of *Caballeronia* in PMAxx-treated soil samples relative to untreated soil samples was unexpected*.* The PMAxx-treated soil samples underwent vigorous, intermittent shaking during the light treatment step, which was not performed on the soil samples for standard qPCR. This mechanical agitation might have dislodged more cells from the soil, leading to higher detectable cell counts in PMAxx-treated soil samples. (Ramsay 1984). The observed higher abundance in PMA-treated soil samples at 0-bug and 5-bug treatments could also be attributed to discrete soil sampling, which may fail to capture the fine-scale spatial variability in the distribution of soil bacteria (Morris, 1999).

Furthermore, we ran positive and negative PMAxx controls (soils spiked with live and dead cells) along with each set of the experimental soil samples we treated. The difference in threshold cycle($\Delta$Ct) between PMAxx-treated and untreated soils spiked with live-cell controls was 1.86, 0.24, and 0.36. These values mostly fell within the manufacturer's recommended difference in threshold cycles ($\Delta$Ct) of 0 ± 1 for live cell control, except for one. Prior studies have shown that higher PMAxx concentrations can reduce live-cell signal in certain microbial species due to PMA-induced cytotoxicity and dye penetration into live, but membrane-compromised, cells (Chen, 2024; Tekgül & Adıgüzel, 2025). The difference in threshold cycle ($\Delta$Ct) between PMAxx-treated and untreated soils spiked with dead-cell controls was 2.76, 2.89, and 3.96, corresponding to 85.24%, 86.5%, and 93.57% reduction in quantification of dead cells, respectively. These values were consistently below the manufacturer's recommended difference in threshold cycles ($\Delta$Ct) ≥ 4 for dead cells control (Biotium, California, US). This discrepancy indicates that our modified PMAxx protocol inhibited amplification of a substantial portion of the dead cells, but not all. In complex samples, such as soils, PMAxx efficiency is often reduced by high turbidity and the presence of organic matter, which can lead to incomplete photoactivation due to shading and interfere with dye binding to dead cells (Bae & Wuertz, 2009; Kaur et al., 2025; Tekgül & Adıgüzel, 2025). Soil matrices are dominated by large quantities of extracellular DNA from dead cells, which can also decrease PMAxx effectiveness (Carini et al., 2016; Fittipaldi et al., 2012; Kaur et al., 2025; Seinige et al., 2014). Consequently, our estimates of live cell count may be overestimated.

Overall, while imperfect, our PMAxx work suggests that a substantial fraction of the DHMR copies we measured with traditional DNA extraction and qPCR derived from live *Caballeronia* cells rather than dead *Caballeronia* DNA.


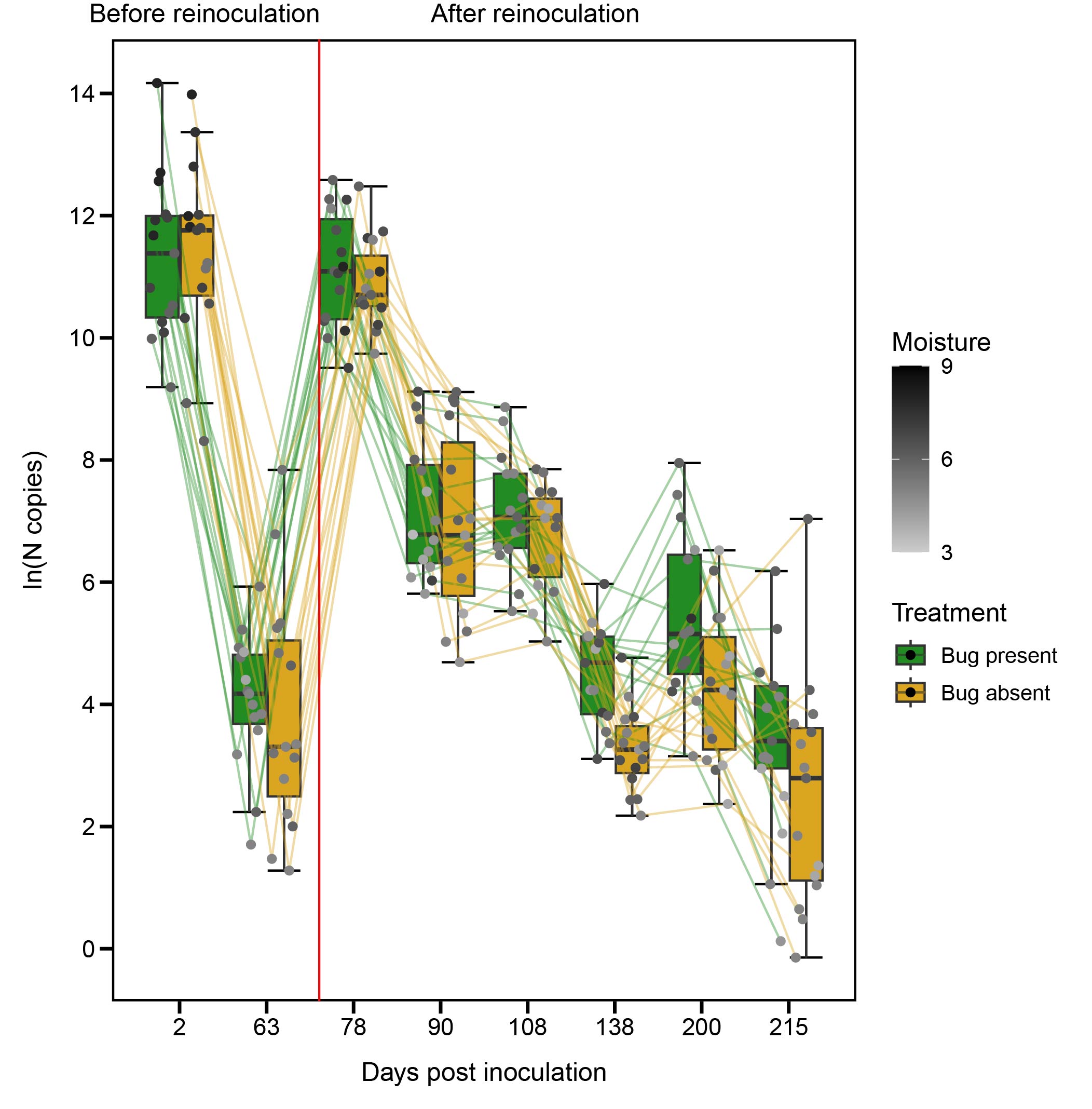


**Supplemental Figure 2:** *Caballeronia* abundance in soil over time in Experiment 2. Each point reports the mean of triplicate titer measurements for an individual replicate microcosm, with lines tracing the trajectory of *Caballeronia* abundance in each microcosm over time. Y-axis reports the titer of *C. grimmiae* in 200 mg of soil. X axis reports the days after the initial inoculation (“Days post inoculation”). Instances where soils were collected over 2-3 days have been set to a single day. Microcosms were reinoculated on Day 76 (red vertical line). The greyscale gradient on the individual data points represents the moisture level of the enclosure at the time of sampling.

**References**

Bae, S., & Wuertz, S. (2009). Discrimination of Viable and Dead Fecal *Bacteroidales* Bacteria by Quantitative PCR with Propidium Monoazide. *Applied and Environmental Microbiology*, *75*(9), 2940–2944. <https://doi.org/10.1128/AEM.01333-08>

Carini, P., Delgado-Baquerizo, M., Hinckley, E.-L. S., Holland‐Moritz, H., Brewer, T. E., Rue, G., Vanderburgh, C., McKnight, D., & Fierer, N. (2020). Effects of Spatial Variability and Relic DNA Removal on the Detection of Temporal Dynamics in Soil Microbial Communities. *mBio*, *11*(1), e02776-19. <https://doi.org/10.1128/mBio.02776-19>

Carini, P., Marsden, P. J., Leff, J. W., Morgan, E. E., Strickland, M. S., & Fierer, N. (2016). Relic DNA is abundant in soil and obscures estimates of soil microbial diversity. *Nature Microbiology*, *2*(3), 16242. <https://doi.org/10.1038/nmicrobiol.2016.242>

Chen, Z. (2024). Enhanced Detection of Viable Escherichia coli O157:H7 in Romaine Lettuce Wash Water Using On-Filter Propidium Monoazide-Quantitative PCR. *Microorganisms*, *13*(1), 34. https://doi.org/10.3390/microorganisms13010034

Fittipaldi, M., Nocker, A., & Codony, F. (2012). Progress in understanding preferential detection of live cells using viability dyes in combination with DNA amplification. *Journal of Microbiological Methods*, *91*(2), 276–289. <https://doi.org/10.1016/j.mimet.2012.08.007>

Fongaro, G., Hernández, M., García-González, M. C., Barardi, C. R. M., & Rodríguez-Lázaro, D. (2016). Propidium Monoazide Coupled with PCR Predicts Infectivity of Enteric Viruses in Swine Manure and Biofertilized Soil. *Food and Environmental Virology*, *8*(1), 79–85. <https://doi.org/10.1007/s12560-015-9225-1>

Guo, F., & Zhang, T. (2014). Detecting the Nonviable and Heat-Tolerant Bacteria in Activated Sludge by Minimizing DNA from Dead Cells. *Microbial Ecology*, *67*(4), 829–836. <https://doi.org/10.1007/s00248-014-0389-2>

Kaur, S., Bran, L., Rudakov, G., Wang, J., & Verma, M. S. (2025). Propidium Monoazide is Unreliable for Quantitative Live–Dead Molecular Assays. *Analytical Chemistry*, *97*(5), 2914–2921. <https://doi.org/10.1021/acs.analchem.4c05593>

Morris, S. (1999). Spatial distribution of fungal and bacterial biomass in southern Ohio hardwood forest soils: Fine scale variability and microscale patterns. *Soil Biology and Biochemistry*, *31*(10), 1375–1386. https://doi.org/10.1016/S0038-0717(99)00047-4

Ramsay, A. J. (1984). Extraction of bacteria from soil: Efficiency of shaking or ultrasonication as indicated by direct counts and autoradiography. *Soil Biology and Biochemistry*, *16*(5), 475–481. <https://doi.org/10.1016/0038-0717(84)90055-5>

Seinige, D., Krischek, C., Klein, G., & Kehrenberg, C. (2014). Comparative Analysis and Limitations of Ethidium Monoazide and Propidium Monoazide Treatments for the Differentiation of Viable and Nonviable Campylobacter Cells. *Applied and Environmental Microbiology*, *80*(7), 2186–2192. <https://doi.org/10.1128/AEM.03962-13>

Tekgül, Z. B., & Adıgüzel, A. (2025). Microbial viability assessment with PMA-qPCR: Challenges, opportunities, and future directions. *Archives of Microbiology*, *207*(12), 343. <https://doi.org/10.1007/s00203-025-04552-4>

Thomas, M. C., Waugh, G., Damjanovic, K., Vanwonterghem, I., Webster, N. S., Negri, A. P., & Luter, H. M. (2025). Development of a quantitative PMA-16S rRNA gene sequencing workflow for absolute abundance measurements of seawater microbial communities. Environmental Microbiome, 20(1), 81. <https://doi.org/10.1186/s40793-025-00741-2>

Nocker, A., Cheung, C.-Y., & Camper, A. K. (2006). Comparison of propidium monoazide with ethidium monoazide for differentiation of live vs. Dead bacteria by selective removal of DNA from dead cells. Journal of Microbiological Methods, 67(2), 310–320. <https://doi.org/10.1016/j.mimet.2006.04.015>

Nocker, A., Sossa-Fernandez, P., Burr, M. D., & Camper, A. K. (2007). Use of Propidium Monoazide for Live/Dead Distinction in Microbial Ecology. Applied and Environmental Microbiology, 73(16), 5111–5117.
